# Assessing the *In Situ* Fertilization Status of Two Marine Copepod Species, *Temora longicornis* and *Eurytemora herdmani*; How Common Are Unfertilized Eggs in Nature?

**DOI:** 10.1371/journal.pone.0112920

**Published:** 2014-11-14

**Authors:** Rachel S. Lasley-Rasher, Andrew M. Kramer, Victoria Burdett-Coutts, Jeannette Yen

**Affiliations:** 1 School of Biology, Georgia Institute of Technology, Atlanta, Georgia, United States of America; 2 Odum School of Ecology, University of Georgia, Athens, Georgia, United States of America; 3 Department of Ocean Science, Memorial University of Newfoundland, St. John’s, Newfoundland and Labrador, Canada; Stazione Zoologica Anton Dohrn, Naples, Italy

## Abstract

We utilized an egg staining technique to measure the *in situ* fertilization success of two marine copepod species, *Temora longicornis* and *Eurytemora herdmani* from May to October 2008 in coastal Maine and correlated fertilization success with environmental conditions in their habitat. *T. longicornis* is a free spawning species that releases eggs into the ambient seawater after mating. In contrast, *E. herdmani* carries eggs in an egg sac until they hatch. The proportion of fertilized eggs within *E. herdmani* egg sacs was significantly higher than the freely spawned clutches of *T. longicornis.* This may be a result of the asymmetrical costs associated with carrying vs. spawning unfertilized eggs. *T. longicornis* frequently laid both fertilized and unfertilized eggs within their clutch. *T. longicornis* fertilization was negatively associated with chlorophyll concentration and positively associated with population density in their local habitat. The fertilization status of *E. herdmani* egg sacs was high throughout the season, but the proportion of ovigerous females was negatively associated with an interaction between predators and the proportion of females in the population. This study emphasizes that, in addition to population level processes, community and ecosystem level processes strongly influence the fertilization success and subsequent productivity of copepods.

## Introduction

In marine food webs, calanoid copepods perform vital ecosystem functions such as trophically connecting phytoplankton productivity to higher-level consumers including many commercially important species [1], [2] affecting carbon export to the deep ocean in the form of sinking fecal pellets [3], [4]. The essential position that copepods hold in marine food webs has prompted investigators to examine factors limiting their productivity by measuring egg production rate [5], [6] in relation to environmental factors such as temperature or food concentration [7], [8]. Additionally, researchers often monitor egg hatching success for a more accurate depiction of potential recruitment but results from these studies frequently yield variable hatching success [9], [10], [11], with the cause rarely determined (but *see* [12], [13]).

Hatching failure in copepod production experiments can be the result of the production of resting eggs (which introduce a time lag into the hatching process), maternal assimilation of toxins and the production of unfertilized eggs. The ecological effect of resting eggs (i.e., fertilized eggs that remain dormant until favorable conditions induce hatching) on copepod population dynamics has received considerable attention in recent decades [14], [15], [16], [17]. Furthermore, the importance of inadequate nutrition [18], [19] and dietary toxins affecting hatching success has been extensively studied [20], [2], [21], [22]. In contrast, very few studies have measured the fertilization status of copepod eggs in the field [12], [13], [23]. Therefore a central question remains as to the importance of fertilization limitation in reducing copepod productivity in nature.

Fertilization limitation occurs when reproductive success is limited by successful mating events [24] and is a well described phenomenon for benthic invertebrates that broadcast spawn their gametes such as sea urchins [25]. Marine copepods have internal fertilization but often occur at low population densities which can hinder mate finding, potentially decreasing population growth [26], [27]. Furthermore, even when encounter rates between potential mates are high, mating frequency and success may be reduced in the presence of predators [28], [29] or abundant heterospecifics [30], [31], [32]. Additionally, recent work suggests that copepod mating success can be reduced when there are abundant males in the population due to increased sexual selection and male-male competition [33]. Finally, eager mates can be further limited by their own physiology; males of many copepod species have a low fertilization capacity [34], [35]. Therefore, the factors that disrupt copepod mating success span individual, population and community scales.

During a successful mating event, a male deposits a spermatophore onto a female’s urosome. The spermatophore empties into the female’s genital atrium where eggs are fertilized before being extruded [3]. Coastal marine copepods such as *Temora longicornis* and *Eurytemora herdmani* lack the long-term sperm storage abilities of offshore copepods [36] and must, therefore, mate repeatedly throughout their lifetime to continue extruding fertilized eggs. The manner in which females extrude their eggs depends on their egg production strategy. There are free spawning species that release their eggs into the surrounding seawater and brooding species that carry their eggs in an egg sac until they hatch [3] as well as a recently-described strategy found in ancestral copepods in which females adhere their eggs to a hard substrate [8]. We focus here on brooding versus free-spawning strategies as these are the most common strategies found in calanoid copepods [3]. Brooding species often have lower egg production rates than spawning species. However, the survival rate is much higher for brooded vs. spawned eggs (reviewed by KiØrboe and Sabatini [37]) with the mortality rate of spawned eggs being as high as 99% at certain times of the year due to predation, sedimentation and cannibalism [38]. Females that brood their eggs can avert such high egg loss but carrying egg sacs often increases vulnerability to predation through increased conspicuousness and reduced escape behavior [39], [28]. If copepods commonly produce unfertilized eggs in nature the cost would likely be much higher for egg brooding species.

Until recently, assessing fertilization required staining live eggs within an hour of egg deposition [12]. Additionally, staining the eggs required multiple steps, such as rinsing several times with fresh seawater. These steps increased egg loss during processing and made it difficult to analyze several samples simultaneously. More recently, this technique has been modified making it possible to stain preserved eggs in a single step, thus greatly increasing the number of samples that can be processed simultaneously [23]. This allows one to process a large number of samples, extending the temporal scale over which comparisons can be made. Comparisons of samples from different times of the year allow determination of the factors associated with fertilization rates in the field.

In this study, we compare the *in situ* fertilization status of egg clutches produced by *T. longicornis,* a broadcast spawner, to *E. herdmani,* a brooder. These marine copepods live together in coastal waters of Maine. We asked: 1) What is the prevalence of unfertilized eggs within the clutches of *T. longicornis* and *E. herdmani*? 2) What population and community level factors correlate with periods of fertilization limitation? By addressing these questions for species with two contrasting spawning strategies we can learn how fertilization dynamics differ for organisms that incur unequal costs due to egg production.

## Methods

### Survey methods

Our survey was conducted from the dock at the Darling Marine Center located in the Damariscotta River estuary, Walpole, Maine (43°56′ N, 69°35′ W). Our study site is part of land is owned by the University of Maine. Our sampling methods did not involve contact with endangered species or vertebrate organisms. Therefore, no specific permission was required for our survey.

The Damariscotta River estuary is a glacially carved drowned river valley that receives very little freshwater input from the Damariscotta Lake. Therefore, conditions in the estuary are very similar to the surrounding coastal waters. Furthermore, the estuary is vertically and horizontally well mixed [40].

We conducted monthly zooplankton surveys from May to October 2008. Our survey consisted of five daily samples being collected within one week, followed by three weeks of no sampling. The water depth at our sampling location is 10–15 m (at low and high tide respectively). Two and a half hours before sunrise during an ebb tide, a plankton net (1 m in diameter) with a mesh size of 250 µm and a flowmeter affixed to the center of the net opening was slowly lowered and suspended in the middle of the water column for approximately 10–15 min. The average sampled volume was 26 m^−3^±3.4 (mean ± SE). We chose to keep our net suspended in the middle of the water column (as opposed to conducting vertical tows) to maximize the volume of water sampled. Additionally, because the sampled animals are those being swept out of the estuary with the tidal current, we think this method yields a representative sample of interacting animals which is important for calculating population density and sex ratio.

At the end of the sampling period, the cod end was emptied into a bucket containing 15 l of surface seawater to dilute the zooplankton. The net was then rinsed and placed back in the water for a second collection. The purpose of dual collections was to maximize the number of females caught each day while minimizing the opportunity for animals to mate under crowded conditions within the sampling net. Gravid *T. longicornis* and *E. herdmani* females bearing ripe oocytes were removed from the samples within 20 minutes of each collection using a wide-bore pipette. Females were placed in individual 5 mL well plates containing ambient seawater and placed in a temperature-controlled room set to ambient temperature (11–18°C). The number of females incubated per monthly sampling period ranged from 31–57 females for *T. longicornis* and 11–19 for *E. herdmani*. After females were separated, the remaining portion of the zooplankton samples were preserved in 4% buffered formalin.

### Environmental sampling

Measurements of temperature and chlorophyll concentration were obtained from the Perry Phytoplankton and Optics Lab at the Darling Marine Center. Samples were collected from the dock two days a week in the morning and extracted in acetone to measure chlorophyll concentration using a Turner Designs fluorometer [41], [42]. Water temperature was measured with a glass, laboratory thermometer. Because temperature and food concentrations were not measured at the exact time of our zooplankton collections, our measurements were inappropriate for addressing small scale temporal differences in these parameters. Therefore, temperature and chlorophyll values were averaged across each sampling day and the 2 days prior. This average more accurately reflects the resolution of our temperature and chlorophyll measurements. Salinity measurements were unavailable during our sampling period. However, Damariscotta River estuary receives little freshwater input and previous surveys of our site show that salinity typically varies less than 3 ppt from May to October [43]. This fluctuation is much less than values shown to affect behavior [44] or reproduction in other copepods species [45], [46].

### Egg collection

Over the course of 6 h, females were visually inspected for the presence of eggs every 50–60 min. For *E. herdmani*, which produce eggs inside an egg sac, the female with attached egg sac were placed in 96 well plates and preserved with 4% buffered formalin. Plates were wrapped in parafilm and vacuum sealed in plastic bags to prevent evaporation during storage.

For *T. longicornis,* which spawns their eggs freely, eggs were counted and collected from the bottom of the wells every 50–60 min, but females were allowed to remain in wells for the remainder of the incubation period (6 h). Using a drawn Pasteur pipette, eggs were transferred to a 96 well plate and preserved in 4% buffered formalin. All eggs from one female were combined into one well, plates were wrapped in parafilm and vacuum sealed in plastic bags to prevent evaporation during storage.

The small volume (5 mL) used for copepod incubation was necessary to ensure that we could scan the entire volume each hour and collect eggs from multiple females. To confirm that egg production was not altered by the small volume and frequent disturbances experienced during our incubations, we conducted supplemental experiments comparing egg production rate and hatching success of *T. longicornis* females in 5 mL well plates vs. 200 mL bottles. Field-caught females were housed in 20 l buckets with males (2∶1, male:female) and fed *Rhodomonas lens* and *Tetraselmis spp.* at non-limiting concentrations. After 3 days, healthy, gravid females were selected from buckets and randomly assigned to their respective treatments. In the 5 mL treatment, females were subjected to the same disturbances as our survey incubations. Females were inspected every hour (for 6 h) under a microscope and eggs were removed with a pipette and placed into a separate well for 48 h. In the 200 mL treatments, females were left undisturbed for 6 h, upon which the contents of the bottle were poured into a dissecting dish, females were removed, and the dish was covered for 48 h. Following the 48 h incubations, acetic acid was added to dishes and well plates to stain nauplii which were immediately counted. We found no difference between the number of eggs produced (*t*-test, *t* = 1.1, df = 29, *p = *0.30) or hatching success (*t*-test, *t* = 0.58, df = 29, *p = *0.56) in small vs. large volumes. We therefore, conclude that the frequent disturbance and small volume had a negligible effect on *T. longicornis* reproduction. The proportion of fertilized eggs within *E. herdmani* egg sacs was consistently high throughout the season (*see* Results), indicating the incubation methods were sufficient for this species and that supplemental experiments were unnecessary.

### Fertilization analysis

The fertilization status of preserved clutches was analyzed within 6 months of collection. To begin analysis, eggs were transferred to a depression slide containing 50 µL of 50 µg mL^−1^ of the vital fluorescent stain, Hoechst 33342 dissolved in 15 ppt filtered seawater. We used this low salinity seawater to induce an osmotic stress on the egg and facilitate stain penetration. Eggs were carefully transferred with minimal media using a drawn Pasteur pipette and no more than 30 eggs slide^−1^ were analyzed. Approximately 50 µL of seawater was transferred along with eggs, yielding a final concentration of approximately 25 µg of fluorescent stain mL^−1^. Slides were covered with a coverslip and placed on a wet paper towel in a sealed plastic container to prevent evaporation and incubated for 10 minutes in the dark at 12°C. Slides were observed at 40x under an Olympus BX-FLA fluorescence microscope with an ultraviolet laser (364 nm *λ*). For each egg, the presence of a male and female pronuclei or the presence of a single female pronuclei were observed as a proxy for a fertilized or unfertilized egg, respectively [12], [13].

Misidentification of eggs can occur in two ways. A polar body could be mistaken as a male’s pronuclei and the egg would be scored as fertilized when it was actually unfertilized. Secondly, a fertilized egg that was preserved during fusion could be mistaken as an unfertilized egg. Therefore, preliminary observations were conducted on freshly-laid eggs and monitored for 4 h at 18°C (the highest temperature observed during the survey). For fertilized eggs, the male and female pronuclei were still visible over an hour after egg deposition. This verifies the presence of two nuclei as a good proxy for fertilization within our samples because all eggs were preserved within an hour of deposition. For unfertilized eggs, only one pronuclei was observed (no visible polar body) and remained un-changed for 4 h. To further verify that eggs with just one pronuclei were unfertilized, oocytes were dissected from 5 gravid females (of both species) for comparison. Unfertilized eggs appeared identical to the dissected oocytes as observed by Ianora et al. [12].

### Zooplankton analysis

Our aim was to obtain an ecological snapshot of the zooplankton community to determine what factors correlated with *in situ* fertilization success in *T. longicornis* and *E. herdmani.* We chose variables that are known to affect aspects of copepod reproduction through effects on mating behavior such as population density, relative species abundance, predator presence and sex ratio [47]. We also included variables that affect copepod egg production such as chlorophyll concentration, and temperature. For each sample the gender and species of adult copepods were identified, allowing for estimates of population densities, sex ratios and relative species abundance. These values were corrected for the number of females removed for fertilization analysis. No attempt was made to identify immature or naupliar stages because our net did not allow for quantitative sampling of these animals and because our main focus was on factors that could affect fertilization success. Non-copepod zooplankton such as polychaetes, predatory mysid shrimp and gelatinous zooplankton were identified to genus or species when possible.

The number of *E. herdmani* females carrying extruded egg sacs (i.e. ovigerous) vs. gravid females without egg sacs also was determined. In this case, we define gravid as females that possess dark ripened ovaries which represents a female’s potential to be mated. It is necessary to include only gravid females with dark oocytes to minimize variation associated with oocyte development time or latency time (i.e., the time between nauplii hatching and the extrusion of a new egg sac); both of which are sensitive to environmental conditions [45]. The proportion of ovigerous females out of the total number of females is commonly used as a proxy for reproductive females [48], [49], [50]. To correct for any females that lost their egg sac during preservation or handling, we searched for detached egg sacs within the samples to obtain an egg sac volume^−1^ estimate. Very few detached egg sacs (5 egg sacs out of 60 zooplankton samples) were found. Supplemental observations indicated that egg sacs remain attached to *E. herdmani* females during the preservation process and persist despite intense physical disturbances (such as vortexing the sample media). Additionally, when trying to detach egg sacs from females (for egg staining purposes) the egg sacs often had to be removed from the females using straight point dissecting needle.

### Statistical analysis

All statistical tests were performed using the software R [51]. To determine differences between the number of fertilized and un-fertilized eggs within *T. longicornis* vs. *E. herdmani* clutches, we fit a generalized linear model (GLM) with logit-link function for quasibinomial distribution to account for significant overdispersion (due to high variance in the number of eggs produced) with species as our explanatory variable [52]. For both *E. herdmani* and *T. longicornis,* we determined what factors were associated with fertilization success (number of fertilized and un-fertilized eggs) by fitting a generalized linear mixed model (GLMM) with a logit-link function for binomial distribution using the R package ‘lme4’ (http://lme4.r-forge.r-project.org/). We considered population density, sex ratio, relative species abundance, predator presence, temperature, chlorophyll concentration and tidal height as fixed explanatory variables (along with a priori selected interactions, *see* Table 1). We selected these variables which we predicted could affect copepod fertilization through alterations in copepod health, egg production, behavior or encounter rates. We used calendar day as a random variable to account for variation within the data associated with the sampling period and because an unequal number of clutches that were analyzed each day. All zooplankton abundance data were pooled by sampling date. The benefit of comparing measurements pooled by day is that this lessens the error associated with sampling a highly patchy environment. In cases where interactions between explanatory variables were found to be significant, we conducted a Pearson’s test to detect correlations between variables. Additionally we determined months in which *E. herdmani* and *T. longicornis* sex ratios were significantly skewed by conducting a one-sample *t*-test to compare the proportion of females in the population to the expected proportion of 0.5 (equal sex ratio).

**Table 1:** Description of a priori selected interaction terms included in generalized linear mixed models assessing the effect of ecological and environmental parameters on *Temora longicornis* and *Eurytemora herdmani* fertilization success.

Interactions	Primary justification for inclusion in model
Density×sex ratio	Low densities may have more of an effect when the sex ratio is skewed (and vice versa)
Density×chlorophyll concentration	Higher food concentration likely supports higher copepod densities.
Density×relative abundance	A combination of a low population density and low relative abundance may increase the occurrence of heterospecific mating events (gamete wastage).
Density×mysid abundance	Mysids may reduce copepod densities through predation.
Sex ratio×mysid abundance	Mysids may skew copepod sex ratio by preying differentially on a gender.
Sex ratio×relative abundance	A combination of a male skewed sex ratio and low relative abundance may increase the occurrence of heterospecific mating events (gamete wastage).
Temperature×chlorophyll concentration	A combination of high temperatures and high food concentration may increase egg production rate, giving females less time be mated between spawning events.

We found chlorophyll concentration to be significantly associated with *T. longicornis* fertilization success (s*ee* Results, Table 2). We were interested in whether or not this relationship is mediated through changes in egg production rate. To analyze differences in the number of eggs produced throughout our survey, we used a zero-inflated Poisson regression with chlorophyll concentration as an explanatory variable [52]. We specified a Poisson error structure given the fact that our data are counts [52]. Furthermore, given the large number females that did not produce eggs, the zero-inflated Poisson regression was appropriate because it takes into account the binary of the component of the data (i.e., females that produced or did not produce eggs) as well as numerical component of the data (i.e., the number of eggs produced).

**Table 2:** Results from generalized linear mixed models (GLMMs) describing factors associated with fertilization in two dominant marine copepod species, *Temora longicornis* and *Eurytemora herdmani.*

Species	Term	Coefficient	St. Error	p-value
*Temora longicornis* proportionof fertile eggs within clutch	Intercept	−0.99	1.27	0.44
	Population density	1.58	0.71	0.025*
	Sex ratio	−1.67	2.46	0.49
	Chlorophyll concentration	−0.51	0.17	0.002**
	Relative abundance	−6.91	3.89	0.076
	Mysid abundance	0.24	0.45	0.59
	Temperature	0.15	0.13	0.23
	Tidal height	0.27	0.26	0.3
*Eurytemora herdmani* proportionof fertile eggs within clutch	Intercept	3.41	1.46	0.02
	Population density	−1.2	0.94	0.19
	Sex ratio	3.11	2.45	0.2
	Chlorophyll concentration	−0.17	0.37	0.63
	Relative abundance	0.13	3.11	0.96
	Mysid abundance	1.19	0.82	0.14
	Temperature	0.05	0.17	0.78
	Tidal height	−0.03	0.3	0.91
*Eurytemora herdmani* proportionof egg carrying females within a population	Intercept	0.04	0.63	0.95
	Population density	0.23	0.21	0.25
	Sex ratio	1.44	1.06	0.18
	Chlorophyll concentration	−0.07	0.14	0.59
	Relative abundance	0.33	0.73	0.66
	Mysid abundance	0.83	0.48	0.09
	Temperature	0.04	0.046	0.34
	Tidal height	−0.05	0.06	0.38
	Sex ratio x Mysid	−2.52	1.01	0.01*

Full model outputs from (GLMMs) are shown with only non-significant interactions removed. An asterisk indicates a significant effect. The sign associated with the coefficient indicates whether the relationship between the variables is a positive or negative correlation.

## Results

**Figure 1:**
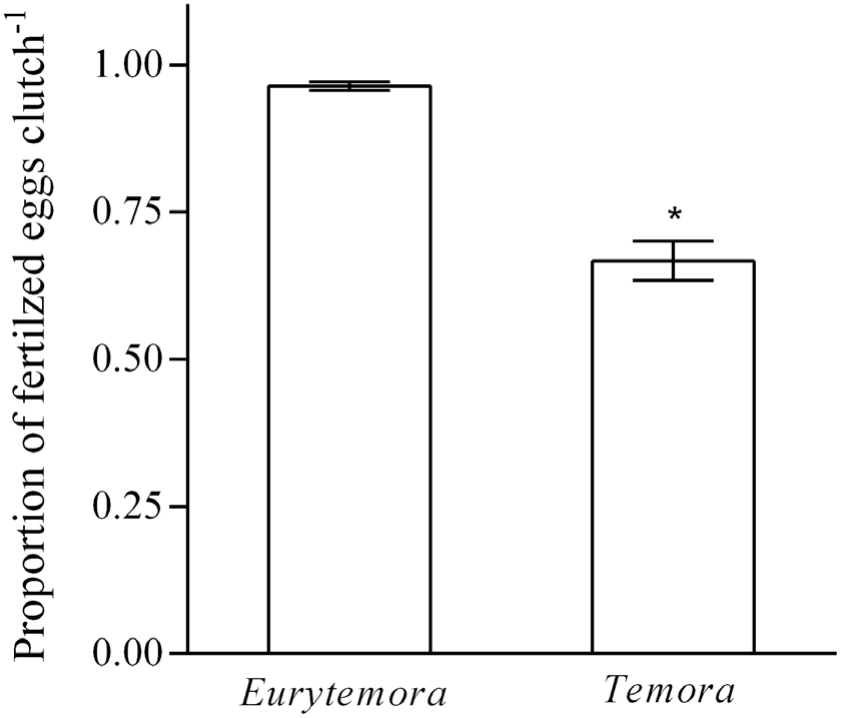
The proportion of fertilized eggs within the clutches of *Eurytemora herdmani* and *Temora longicornis* females (mean ± SE). Females were captured from the field and their clutches stained with a nuclei specific probe (*n* = 68 and *n* = 83 for *E. herdmani* and *T. longicornis,* respectively). Proportion of fertilized eggs were compared using a generalized linear model with a quasibinomial distribution and logit-link function (*F*_1,150_ = 109, *p*<0.001).

**Figure 2:**
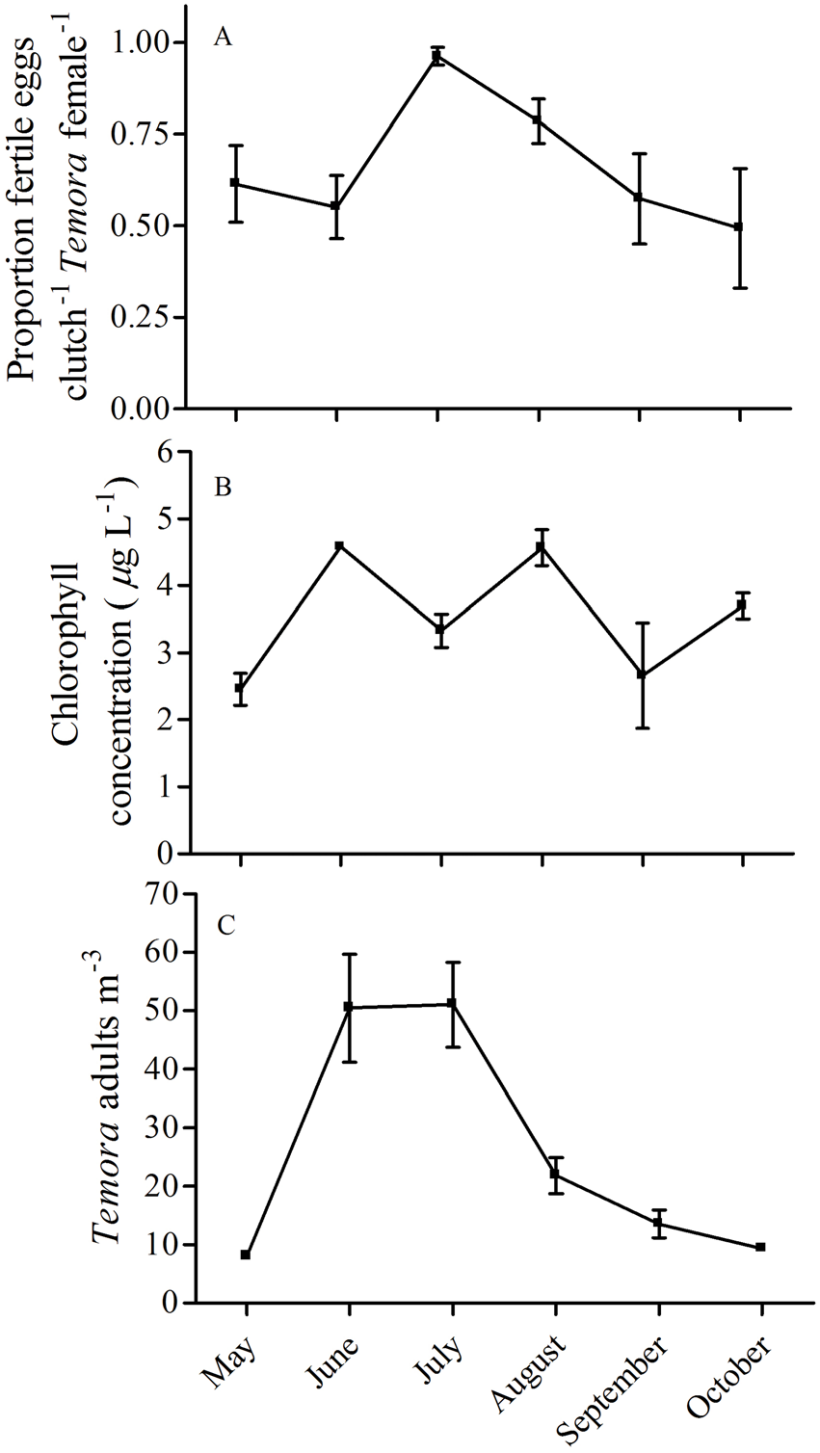
Seasonal dynamics of the (A) fertilization status of *Temora longicornis*, (B) chlorophyll concentration, and (C) population density from the Damariscotta river estuary in coastal Maine, USA. Zooplankton samples were collected during 5 day field surveys conducted at 3 week intervals from May to October 2008 (*n* = 30) in coastal Maine.

Freely spawned clutches of *T. longicornis* contained a significantly lower proportion of fertilized eggs when compared to *E. herdmani* egg sacs (Fig. 1, *F*_1,150_ = 109, *p*<0.001, GLM quasibinomial distribution). Results from our field survey indicated that *T. longicornis* fertilization was negatively associated with chlorophyll concentration (Fig. 2, Table 2, *p* = 0.002, GLMM binomial distribution) and positively associated with population density (Fig. 2, Table 2, *p* = 0.03, GLMM binomial distribution). However, no significant correlation between chlorophyll concentration and population density was detected (Pearson’s correlation test, *r* = 0.13, df = 81, *p* = 0.24). Chlorophyll concentrations ranged from 1.4 to 6.2 µg carbon L^−1^ with minimum and maximum values observed in September and August respectively (Fig. 2B). The zero-inflated Poisson regression model predicting the number of eggs produced by *T. longicornis* in 6 h from chlorophyll concentration values showed a significant positive association between egg production and chlorophyll concentration (*X* ^2^ *= *19.4, df = 2, *p*<0.001). Monthly average population densities of *T. longicornis* ranged from 8 to 51 individuals m^−3^ with minimum densities observed in May and maximum densities observed in June and July (Fig. 2C). There was no significant effect of sex ratio on *T. longicornis* fertilization success (Table 2). The proportion of females within *T. longicornis* populations were not significantly different than 0.5 (equality of sex ratio) during any month in our survey except October when the population was significantly male skewed (one sample *t*-test, *t* = 6.2, df = 4, *p*<0.05).

**Figure 3:**
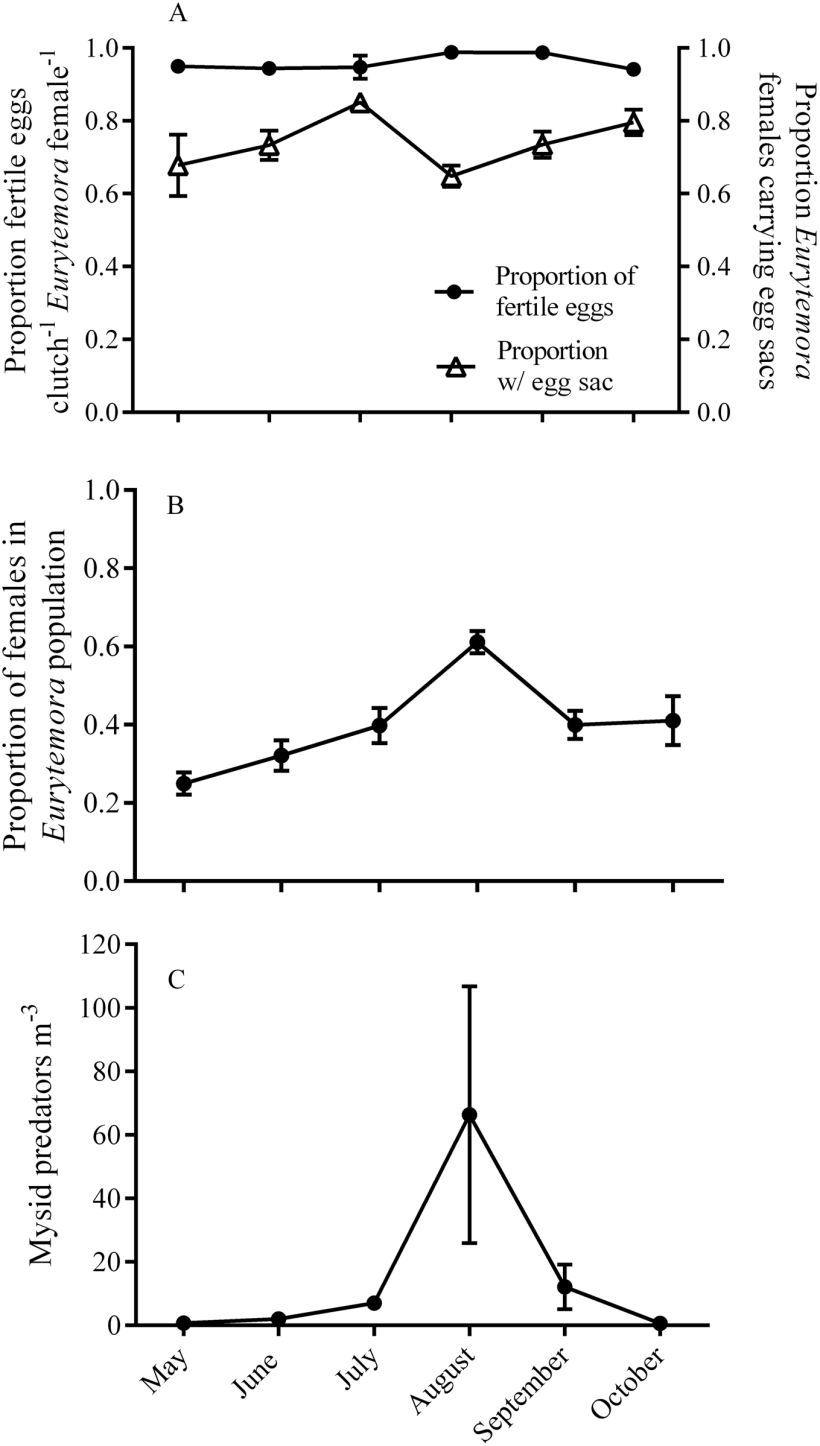
Seasonal dynamics of the (A) fertilization status and ovigery patterns in *Eurytemora herdmani*, (B) the proportion of females within the population, and (C) mysid predator abundance from the Damariscotta river estuary in coastal Maine. Zooplankton samples were collected during 5 day field surveys conducted at 3 week intervals from May to October 2008 (*n* = 30) in coastal Maine.

The proportion of fertilized eggs within *E. herdmani* clutches was very high throughout the season (0.96±0.07, mean ± SE) and did not significantly correlate with any environmental or ecological factors (Fig. 3A, Table 2, p>0.1, GLMM binomial distribution). In contrast, variation in the proportion of egg bearing *E. herdmani* females was negatively correlated with an interaction between the proportion of females in the population and abundant mysid predators (Fig. 3B & 3C, Table 2, *p* = 0.01, GLMM binomial distribution) but neither predators nor proportion female had independent effects. Furthermore, there was a significant correlation between the proportion of females in the population and mysid predator density (Pearson’s correlation test, *r* = 0.53, df = 26, *p = *0.004). The predatory mysid *Neomysis americana* was the most abundant predator throughout our survey. The monthly average mysid density ranged from 0 to 66 individual m^−3^ with minimum and maximum densities observed in May and August, respectively. *E. herdmani* populations were significantly male skewed during three out of the six months surveyed (one sample *t-*test, May, *t* = 8.6, df = 4, *p*<0.05; June, *t* = 4.6, df = 4, *p*<0.05; September, *t* = 2.8, df = 4, *p<*0.05). In August, *E. herdmani* populations were significantly female skewed (one sample *t-*test, *t* = 3.8, df = 4, *p*<0.05). The proportion of females within *E. herdmani* populations were not significantly different than 0.5 (equality of sex ratio) during the months of July and October. Monthly average population densities of *E. herdmani* ranged from 14 to 267 individuals m^−3^, with maximum densities in July and minimum densities in May. However, there was no significant effect of population density on *E. herdmani* fertilization success (Table 2).

## Discussion

*T. longicornis* frequently produced unfertilized eggs within their freely spawned clutches. Conversely, the attached egg sacs of *E. herdmani* rarely contained unfertilized eggs. For an egg brooding female, carrying an egg sac can increase vulnerability to predators and metabolic demands [39], [28]. It is likely that females would be selected to avoid these costs when eggs are unfertilized and the benefit of enhanced offspring survival is not possible.

The cost of eggs going unfertilized is likely lower for *T. longicornis.* Producing eggs is unlikely to increase their susceptibility to predation or increase the cost of swimming because *T. longicornis* spawn their eggs freely. However, females are still investing valuable nutrients in the eggs. Why doesn’t *T. longicornis* hold on to their eggs or resorb them? Oosorption, the process of re-absorbing mature eggs to conserve nutrients during times of starvation or to maintain a constant supply of newly matured eggs, has been well documented in insects [53]. Egg resorption also has been demonstrated in the marine copepod, *Calanus finmarchicus* in response to days of starvation [54]. However, this process has never been demonstrated to occur in response to lack of mating in copepods, which may in part be explained by the short time window some copepod species can produce eggs. Even under optimal laboratory conditions, *T. longicornis* females can only produce eggs for approximately 18 d [55] which is shorter than the egg production time of *Eurytemora affinis* females (approximately 42 d under optimal conditions, [45]. Therefore, it may be a better strategy for *T. longicornis* to continue to produce high quality eggs during this time window, even when successful mating events are rare.

Field surveys that have determined the fertilization status of individual eggs are rare (but *see* [12], [23]. Ianora et al. [12] collected and subsequently incubated *T. stylifera* females from the Gulf of Naples and found that, on average, females produced clutches that were comprised of 60% fertilized eggs. This is comparable to the average fertilization status observed here for *T. longicornis* clutches (i.e., 67% ±3% fertilized, mean ± SE) across the survey period. In contrast, Zirbel et al. [23] discovered that unfertilized eggs made up a very small percentage of the total eggs collected from pump samples taken from the water column in Dabob Bay, Washington. This is likely due to the fact that un-fertilized eggs can disintegrate shortly after deposition (within hours to days [13]) making them more difficult detect in the water column. Additionally, two out of the four species considered by Zirbel et al. [23] possess seminal receptacles, and can store sperm for life [36]. Together these studies suggest that fertilization dynamics may be specific to a particular species or set of environmental conditions. Nonetheless, they highlight the importance of analyzing the fertilization status of individual eggs in order to address the prevalence and causes of fertilization limitation for copepod species.

During our survey, female *T. longicornis* produced high proportions of unfertilized eggs within their clutches at certain times of the year, yet, nearly all females produced at least one fertilized egg. This suggests that a female’s fertilization status is not binary and that females continue to lay eggs after spermatozoa within their genital atrium is depleted. These results corroborate recent findings that fertilization limitation is an important factor constraining population growth in *T. longicornis* [33]. Unfertilized eggs were more common for *T. longicornis* at lower population densities and higher food concentrations. The positive association between population density and fertilization success is likely due to an increase in mate-encounter rates [26], [27]. The mechanism responsible for chlorophyll concentration’s negative association with *T. longicornis* fertilization is less clear. If the elevated chlorophyll levels were caused by low quality foods, this could negatively affect fertilization. Some dinoflagellate species have been shown to decrease spermatophore quality and subsequent fertilization success in the congener, *T. stylifera* [56], [57]. Furthermore, three of the dinoflagellate species identified as having harmful effects on fertilization [57] have been isolated from nearshore waters of the northeastern US coast (https://ncma.bigelow.org). However, our survey was not designed to assess the quality of foods, so this hypothesis remains untested. In contrast to fertilization success, the number of eggs produced by *T. longicornis* during 6 h incubations was positively associated with chlorophyll concentration (*X* ^2^ *= *19.4*;* df = 2; *p*<0.001; zero-inflated regression). It is important to note that our incubations were not long enough to control for diel patterns in egg production rate thus, these results should be interpreted cautiously. Nonetheless, *T. longicornis* can produce approximately 30–75 fertilized eggs after one mating event [55]. Copepod egg production can become food-limited in nature [58], [59] and chlorophyll concentrations dipped to food-limiting levels during our survey (i.e. 2 µg L^−1^) [60]. Therefore, it is plausible that increases in food concentration may result in more unfertilized eggs because it speeds up egg production rate and decreases the amount of time that females have to mate between spawning events. This is especially true for coastal marine copepods that lack long-term sperm storage [36] such as *T. longicornis* (and *E. herdmani*). However, this hypothesis cannot be addressed with these data. Future studies addressing the effects of egg production rate on fertilization are necessary to determine whether this correlation exist.

*E. herdmani* consistently had a high proportion of fertilized eggs within their eggs sacs throughout the season. However, there was significant variation in the proportion of ovigerous *E. herdmani* females in the population which was negatively correlated with an interaction between mysid predator abundance and the proportion of females in the population. Importantly, neither factor had independent effects on the number of egg-bearing females.

The number of ovigerous females in a population is affected by egg production dynamics (i.e. rate of egg production and release), mating success as well as differential mortality between ovigerous and non-ovigerous females. Female copepods may alter egg production in response to predators and mating opportunities, which could have reduced the proportion of ovigerous females. In a mesocosm experiment, Heuschele et al. [61] found the ratio of egg-bearing to non-egg-bearing *E. affinis* females was reduced in the presence of mysid predators, while the number of mated females was unaffected. Mysid predators also had no effect on density or sex ratio of the mesocosm population, therefore, the authors attributed their results to females delaying reproduction in the presence of predators or shedding egg sacs [61]. There is circumstantial evidence that females of some copepod species may delay the extrusion of egg sacs until becoming fertilized which would result in longer latency times (i.e. the time from nauplii hatching until a new egg sac is extruded) and subsequently, fewer ovigerous females. Williamson & Butler [62] observed that egg production rate in *Diaptomus paliidus* varied with the density of males suggesting that females were delaying clutch production when mating events were rare [62]. Furthermore, Devreker et al. [45] observed exceptionally long latency times in *E. affinis* females that lost their spermatophores after mating, suggesting that sperm limitation could have caused the females to delay clutch production [45].

An alternative explanation is that under conditions of abundant mysids and female-skewed sex ratios *E. herdmani* mating success is disrupted, thus reducing the number of egg-bearing females in the population. In manipulative experiments, *E. herdmani* reduced mating frequency and offspring production in the presence of a mysid predator cue even in the absence of an actual predator [29]. Furthermore, another egg-bearing species, *Oithona davisae,* suffer reduced fertilization when populations are strongly female-skewed [34]. It is unclear whether or not mysids are responsible for skewing the sex ratio through male ingestion. Results from small incubations and large mesocom experiments, have failed to find evidence for mysids preferentially preying on male *Eurytemora spp.* [61], [29].

Finally, differential mortality of ovigerous females may be driving the observed patterns. There is evidence that ovigerous females are more vulnerable to predation through increased conspicuousness and reduced escape behavior [39], [28]. However, results from a mesocosm experiment suggest that mysids do not preferentially remove ovigerous females [61]. It is unclear whether or not mysids preferentially removed ovigerous females during our survey. However, is important to note that we did not observe an independent effect of mysids on the ratio of ovigerous females which suggests that this unlikely played a role in our observed results.

In this study, we determined the *in situ* fertilization status of *E. herdmani* and *T. longicornis* females. This study provides needed field observations of the fertilization status of copepods in nature. We found reproduction to be limited through different mechanisms for these species, fertilization limitation in *T. longicornis* versus reduced ovigery in *E. herdmani*. Factors that limit copepod reproduction (i.e., food abundance, predator presence, population density and sex ratio) span individual, population and community level scales. This research highlights the need to look beyond demography (i.e., population density and sex ratio) when addressing constraints on copepod population growth.

## References

[1] Runge JA (1988) Should we expect a relationship between primary production and fisheries - The role of copepod dynamics as a filter of trophic variability. Hydrobiologia 167: 61–71.

[2] Irigoien X, Harris RP, Verheye HM, Joly P, Runge J, et al (2002) Copepod hatching success in marine ecosystems with high diatom concentrations. Nature 419: 387–389. 12353032 10.1038/nature01055

[3] Mauchline J (1998) The biology of calanoid copepods. Adv Mar Biol 33.

[4] Dagg MJ, Urban-Rich J, Peterson JO (2003) The potential contribution of fecal pellets from large copepods to the flux of biogenic silica and particulate organic carbon in the Antarctic Polar Front region near 170 degrees W. Deep Sea Res., Part II. 50: 675–691.

[5] Uye SI (1982) Population dynamics and production of *Acartia clausi,* Giesbrecht (Copepoda, Calanoida) in inlet waters. J Exp Mar Biol Ecol 57: 55–83.

[6] Huntley ME, Lopez MDG (1992) Temperature-dependant production of marine copepods - A global synthesis. Am Nat 140: 201–242. 19426057 10.1086/285410

[7] Boyer S, Bouvy M, Bonnet D (2013) What triggers *Acartia* species egg production in a Mediterranean lagoon? Estuarine Coastal Shelf Sci 117: 125–135.

[8] Brugnano C, Guglielmo L, Ianora A, Zagami G (2009) Temperature effects on fecundity, development and survival of the benthopelagic calanoid copepod, *Pseudocyclops xiphophorus.* . Mar Biol 156: 331–340.

[9] Devreker D, Souissi S, Seuront L (2005) Effects of chlorophyll concentration and temperature variation on the reproduction and survival of *Temora longicornis* (Copepoda, Calanoida) in the Eastern English Channel. J Exp Mar Biol Ecol 318: 145–162.

[10] Dutz J, van Beusekom JEE, Hinrichs R (2012) Seasonal dynamics of fecundity and recruitment of *Temora longicornis* in the Baltic Sea. Mar Ecol Prog Ser 462: 51–66.

[11] Wesche A, Wiltshire KH, Hirche HJ (2007) Overwintering strategies of dominant calanoid copepods in the German Bight, southern North Sea. Mar Biol 151: 1309–1320.

[12] Ianora A, Dicarlo BS, Mascellaro P (1989) Reproductive biology of the planktonic copepod *Temora stylifera.* . Mar Biol 101: 187–194.

[13] Ianora A, Poulet SA (1993) Egg viability in the copepod *Temora stylifera.* . Limnol Oceanogr 38: 1615–1626.

[14] Belmonte G, Pati AC (2007) Hatching rate and diapause duration in eggs of *Paracartia latisetosa* (Copepoda: Calanoida). J Plankton Res 29: I39–I47.

[15] Hairston NG, Vanbrunt RA, Kearns CM, Engstrom DR (1995) Age and survivorship of diapausing eggs in a sediment egg bank. Ecology 76: 1706–1711.

[16] Marcus NH (1996) Ecological and evolutionary significance of resting eggs in marine copepods: Past, present, and future studies. Hydrobiologia 320: 141–152.

[17] Castellani C, Lucas IAN (2003) Seasonal variation in egg morphology and hatching success in the calanoid copepods *Temora longicornis, Acartia clausi* and *Centropages hamatus.* . J Plankton Res 25: 527–537.

[18] Jonasdottir SH, Gudfinnsson HG, Gislason A, Astthorsson OS (2002) Diet composition and quality for *Calanus finmarchicus* egg production and hatching success off south-west Iceland. Mar Biol 140: 1195–1206.

[19] Tang KW, Taal M (2005) Trophic modification of food quality by heterotrophic protists: species-specific effects on copepod egg production and egg hatching. J Exp Mar Biol Ecol 318: 85–98.

[20] Ban SH, Burns C, Castel J, Chaudron Y, Christou E (1997) The paradox of diatom-copepod interactions. Mar Ecol Prog Ser 157: 287–293.

[21] Ianora A, Miralto A, Poulet SA, Carotenuto Y, Buttino I, et al (2004) Aldehyde suppression of copepod recruitment in blooms of a ubiquitous planktonic diatom. Nature 429: 403–407. 15164060 10.1038/nature02526

[22] Ianora A, Miralto A (2010) Toxigenic effects of diatoms on grazers, phytoplankton and other microbes: a review. Ecotoxicology 19: 493–511. 19924531 10.1007/s10646-009-0434-y

[23] Zirbel MJ, Miller CB, Batchelder HP (2007) Staging egg development of marine copepods with DAPI and PicoGreen (R). Limnol Oceanogr: Methods 5: 106–110.

[24] Gerritsen J (1980) Sex and parthenogenesis in sparse populations. Am Nat 115: 718–742.

[25] Levitan DR, Sewell MA, Chia FS (1992) How distribution and abundance influence fertilization success in the sea urchin *Stongylocentrotus franciscanus.* . Ecology 73: 248–254.

[26] Choi KH, Kimmerer WJ (2008) Mate limitation in an estuarine population of copepods. Limnol Oceanogr 53: 1656–1664.

[27] Choi KH, Kimmerer W (2009) Mating success and its consequences for population growth in an estuarine copepod. Mar Ecol Prog Ser 377: 183–191.

[28] Maier G, Berger I, Burghard W, Nassal B (2000) Is mating of copepods associated with increased risk of predation? J Plankton Res 22: 1977–1987.

[29] Lasley-Rasher RS, Yen J (2012) Predation risk suppresses mating success and offspring production in the coastal marine copepod, *Eurytemora herdmani.* . Limnol Oceanogr 57: 433–440.

[30] Jacoby CA, Youngbluth MJ (1983) Mating behavior in 3 species of *Pseudodiaptomus* (Copepoda, Calanoida). Mar Biol 76: 77–86.

[31] Thum RA (2007) Reproductive interference, priority effects and the maintenance of parapatry in *Skistodiaptomus* copepods. Oikos 116: 759–768.

[32] Goetze E, Kiørboe T (2008) Heterospecific mating and species recognition in the planktonic marine copepods *Temora stylifera* and *T. longicornis.* . Mar Ecol Prog Ser 370: 185–198.

[33] Ceballos S, Sichlau MH, Heuschele J, Kiørboe T (2014) Low fertilization rates in a pelagic copepod caused by sexual selection? J Plankton Res 36: 736–742.

[34] Kiørboe T (2007) Mate finding, mating, and population dynamics in a planktonic copepod *Oithona davisae*: There are too few males. Limnol Oceanogr 52: 1511–1522.

[35] Ceballos S, Kiørboe T (2011) Senescence and sexual selection in a pelagic copepod. PLoS ONE 6(4). 10.1371/journal.pone.0018870 PMC3077418 21533149

[36] Ohtsuka S, Huys R (2001) Sexual dimorphism in calanoid copepods: morphology and function. Hydrobiologia 453: 441–466.

[37] Kiørboe T, Sabatini M (1994) Reproductive and life cycle strategies in egg-carrying cyclopoid and free-spawning calanoid copepods. J Plankton Res 16: 1353–1366.

[38] Peterson WT, Kimmerer WJ (1994) Procusses controlling recruitment of the marine calanoid copepod *Temora longicornis* in Long Island Sound - Egg production, egg mortality, and cohort survival rates. Limnol Oceanogr 39: 1594–1605.

[39] Magnhagen C (1991) Predation risk as a cost of reproduction. Trends Ecol Evol 6: 183–185. 21232452 10.1016/0169-5347(91)90210-O

[40] McAlice C (1977) A preliminary oceanographic survey of the Damariscotta River estuary, Lincoln County, Maine. Maine Seagrant Technical Report.

[41] Parsons TR, Maita Y, Lalli CM (1984) A Manual of Chemical and Biological Methods for Seawater Analysis. Oxford: Pergamon Press.

[42] UNESCO (1994) Intergovernmental Oceanographic Commission Manuals and Guides. 29. Available: http://www.jodc.go.jp/info/ioc_doc/html/manuals.htm. Accessed 15 April 2013.

[43] Thompson BP (2006) Temporal and spatial variability of phytoplankton biomass in the Damariscotta River estuary, Maine, USA. M.Sc. Thesis, University of Maine.

[44] Seuront L (2006) Effect of salinity on the swimming behaviour of the estuarine calanoid copepod *Eurytemora affinis.* . J Plankton Res 28: 805–813.

[45] Devreker D, Souissi S, Winkler G, Forget-Leray J, Leboulenger F (2009) Effects of salinity, temperature and individual variability on the reproduction of *Eurytemora affinis* (Copepoda; Calanoida) from the Seine estuary: A laboratory study. J Exp Mar Biol Ecol 368: 113–123.

[46] Holste L, John MA, Peck MA (2009) The effects of temperature and salinity on reproductive success of *Temora longicornis* in the Baltic Sea: a copepod coping with a tough situation. Mar Biol 156: 527–540.

[47] Kiørboe T (2006) Sex, sex-ratios, and the dynamics of pelagic copepod populations. Oecologia 148: 40–50. 16425044 10.1007/s00442-005-0346-3

[48] Hopkins CCE (1982) The breeding biology of *Euchaeta norvegica* (Boeck) (Copepoda, Calanoida) in Loch Etive, Scotland - Assessment of breeding intensity in terms of seasonal cycles in the sex ratio, spermatophore attachment, and egg sac production. J Exp Mar Biol Ecol 60: 91–102.

[49] Uye S, Sano K (1995) Seasonal reproductive biology of the small cyclopoid copepod *Oithona davisae* in a temperate eutrophic inlet. Mar Ecol Prog Ser 118: 121–128.

[50] Kramer AM, Sarnelle O, Knapp RA (2008) Allee effect limits colonization success of sexually reproducing zooplankton. Ecology 89: 2760–2769. 18959313 10.1890/07-1505.1

[51] Team RCD (2011) R: A language and environment for statistical computing. R Foundation for Statistical Computing. Vienna, Austria.

[52] Crawley MJ (2005) Statistics: An introduction using R. West Sussex: Wiley.

[53] Moore PJ, Harris WE, Moore AJ (2007) The cost of keeping eggs fresh: Quantitative genetic variation in females that mate late relative to sexual maturation. Am Nat 169: 311–322. 17243076 10.1086/510687

[54] Niehoff B (2004) The effect of food limitation on gonad development and egg production of the planktonic copepod *Calanus finmarchicus.* . J Exp Mar Biol Ecol 307: 237–259.

[55] Sichlau MH, Kiørboe T (2011) Age- and size-dependent mating performance and fertility in a pelagic copepod, *Temora longicornis.* . Mar Ecol Prog Ser 442: 123–132.

[56] Ianora A, Poulet SA, Miralto A (1995) A comparative study of the inhibitory effect of diatoms on the reproductive biology of the copepod *Temora stylifera.* . Mar Biol 121: 533–539.

[57] Ianora A, Miralto A, Buttino I, Romano G, Poulet SA (1999) First evidence of some dinoflagellates reducing male copepod fertilization capacity. Limnol Oceanogr 44: 147–153.

[58] Kimmerer WJ, Ferm N, Nicolini MH, Penalva C (2005) Chronic food limitation of egg production in populations of copepods of the genus *Acartia* in the San Francisco Estuary. Estuaries 28: 541–550.

[59] Kiørboe T, Kaas H, Kruse B, Mohlenberg F, Tiselius P, et al (1990) The structure of the pelagic food web in relation to water column structure in the Skagerrak. Mar Ecol Prog Ser 59: 19–32.

[60] Durbin EG, Durbin AG, Smayda TJ, Verity PG (1983) Food limitation of production by adult *Acartia tonsa* in Narragansett Bay, Rhode Island. Limnol Oceanogr 28: 1199–1213.

[61] Heuschele J, Ceballos S, Borg CMA, Bjaerke O, Isari S, et al (2014) Non-consumptive effects of predator presence on copepod reproduction: insights from a mesocosm experiment. Mar Biol 161: 1653–1666.

[62] Williamson CE, Butler NM (1986) Predation on rotifers by the suspension-feeding calanoid copepods *Diaptomus pallidus.* . Limnol Oceanogr 31: 393–402.

